# Dysentery

**Published:** 1848-10

**Authors:** 


*Dysentery.*—We learn from various quarters, that this disease, in an epidemic and malignant form, is, at this time, extensively prevalent. It has prevailed, of late, to some extent in this city and vicinity, but in the majority of instances is mild, and easily managed. A few deaths only have occurred. The diseases of the season, generally, in this city, have been less prevalent and severe than usual.

